# Copper Mineral Leaching Mathematical Models—A Review

**DOI:** 10.3390/ma15051757

**Published:** 2022-02-25

**Authors:** Manuel Saldaña, Edelmira Gálvez, Pedro Robles, Jonathan Castillo, Norman Toro

**Affiliations:** 1 Faculty of Engineering and Architecture, Universidad Arturo Prat, Iquique 1110939, Chile; ntoro@ucn.cl; 2 Departamento de Ingeniería Química y Procesos de Minerales, Facultad de Ingeniería, Universidad de Antofagasta, Antofagasta 1270300, Chile; 3 Departamento de Ingeniería Metalúrgica y Minas, Universidad Católica del Norte, Antofagasta 1270709, Chile; egalvez@ucn.cl; 4 Escuela de Ingeniería Química, Pontificia Universidad Católica De Valparaíso, Valparaíso 2340000, Chile; pedro.robles@pucv.cl; 5 Departamento de Ingeniería en Metalurgia, Universidad de Atacama, Copiapó 1531772, Chile; jonathan.castillo@uda.cl

**Keywords:** mathematical models, copper mineral leaching, CFD, leaching kinetics, metal extraction

## Abstract

Mineral leaching is the key unit operation in metallurgical processes and corresponds to the dissolution of metals. The study of leaching is carried out in many areas, such as geology, agriculture and metallurgy. This paper provides an introduction to the theoretical background regarding the mathematical modelling of the leaching process of copper minerals, establishing an overall picture of the scientific literature on technological developments and the generation of representative mathematical and theoretical models, and indicating the challenges and potential contributions of comprehensive models representing the dynamics of copper mineral leaching.

## 1. Introduction

The global trend towards industrialization supports the increasing demand for industrial metals. It is in this context that low-grade and complex ores, old waste deposits related to past mining worksites and other sources have received attention in recent years, with the latest advances in leaching techniques and applications to the leaching of multiple metals making many operations economically feasible. Leaching processes can be defined as the selective removal and/or extraction of metallic values from a mineral, causing a suitable solvent of the leaching agent to percolate into and through a mass of heap or mineral containing the metallic values [1]. Leaching is of great importance in the field of metallurgy since it is frequently used in the extraction of some minerals such as gold, silver and copper.

World copper mine production decreased slightly to an estimated 20 million tons in 2020 from 20.4 million tons in 2019, mainly due to COVID-19 blockages in April and May. World refined copper production increased slightly to an estimated 25 million tons in 2020 from 24.5 million tons in 2019, when the production in several countries was impacted by temporary smelter shutdowns for maintenance and upgrades [2]. Future copper demand projections indicate that the per capita copper in-use stock (IUS) is expected to fall gradually from the reference level of 240 kg/person to its minimum value of 227 kg/person around the year 2032 before peaking at 243 kg/person in 2070 as a result of growth in some end-use sectors and contraction in others. This represents an increase of 1.0% between 2015 and 2070 [3].

Most of the copper minerals existing on the planet correspond to sulfides and a small part of oxides [4]. The mining industry has traditionally operated in two ways: pyrometallurgy in the case of sulfide minerals, consisting of flotation, smelting and electro-refining processes; while hydrometallurgical processes, composed of leaching, solvent extraction and electroextraction processes, work mainly with oxidized minerals [5]. Both mechanisms have proven to be profitable in the industry. Nevertheless, pyrometallurgy processes (mainly used in old foundries) have the disadvantage of being generators of SO_2_ emissions in the atmosphere, causing serious environmental problems [6]. However, it is hoped that the implications of the technological revolution in mining will contribute to mitigating the negative effects of mining on the environment in which it operates [7].

In the present work, a comprehensive analysis of theoretical copper mineral leaching modeling techniques is developed, including industrial application using heap leaching. Heap leaching mainly requires size reduction in order to maximize the leaching minerals interaction and the placement of an impermeable base to prevent leaching loss and the contamination of water bodies [8]. The heap leaching process came into use in the mid-20th century, when the former U.S. Bureau of Mines developed heap leaching technology to recover precious metals from low grade mineral heaps using cyanide solutions, adsorption on activated charcoal and electrowinning recovery [9]; large-scale mining was not used for heap leaching. Large-scale mining has its origins in 1980, when large copper mining projects in Chile and the first large-scale mining projects in the U.S. were developed [10]. Since then, the progress in technology and the development of improvements in the methodology of obtaining minerals by leaching has increased, being applied to different types of minerals, climates and operations of any size [11]. In addition to copper oxides, it is applied to a wide range of minerals, including copper sulfide minerals such as chalcocite [12,13,14,15], covellite [16,17,18,19,20] or chalcopyrite [21,22,23]. Likewise, leaching can be applied to non-metallic minerals such as saltpeter [24,25] or to the recovery of soils [26,27,28]. Leaching is typically used for high grade mineral deposits (or at least that was its widespread use in recent decades), but as large mineral reserves have depleted, its use has become more widespread for deposits of any size because of the minimization of capital costs associated with its application. 

Some applications of leaching for processing metallic minerals are presented in Table 1.

An overview of its industrial application and bibliometric analysis is conducted in Section 2. A systematic overview of mineral leaching models (expect for bioleaching processes) at the theoretical and empirical, laboratory and industrial scale is developed in Section 3, identifying the key factors that drive mineral leaching processes and highlighting the significant variables that optimize the response, contributing to improve the efficiency and effectiveness of industrial leaching applications. Finally, conclusions, challenges and future directions of the copper leaching modeling are presented in Section 4.

## 2. Leaching Process

### 2.1. Overview and Industrial Applications

Leaching is defined as “the treatment of complex substances, like a mineral, with a specific solvent, able to separate its soluble parts from the insoluble ones” [42]. The process is used for the production of a concentrated solution of valuable solid material or to remove an insoluble solid from a soluble material with which it is contaminated. The method used for extraction is largely determined by the proportion of soluble constituent present, its distribution in the solid, the nature of the solid and its’ particle size [43]. If the solute is uniformly dispersed in the solid, material near the surface will dissolve first, leaving a porous structure in the solid residue. Therefore, the solvent will have to penetrate this outer layer before it can reach more solute, and the process will become progressively more difficult. The extraction rate will decrease [43].

The selection of the level of variables to control in an extraction process is influenced by the factors that are responsible for limiting the extraction rate. There are many factors that impact the extraction rate. Among the most important are the size of the particle, the solvent, the temperature and the agitation of the fluid. Then, if the diffusion of the solute through the porous structure of the residual solids is considered to be the main controlling factor, the material should be of a small size so that the distance that the solute must travel is small. Additionally, if the controlling factor is solute diffusion, a high degree of fluid agitation is required.

Then, within the industrial applications of mineral leaching, there is the technology of heap leaching, technology that was developed in the U.S., nonetheless, it was in Chile where this technology was substantially perfected, achieving practical applications on a large scale (mainly in the copper metallurgical industry), and where the hydrometallurgical process is currently the most used. In the hydrometallurgical process, the crushed material is transported (generally through conveyor belts) to the place where the heap will be formed. In this journey, the material is first subjected to irrigation with a water solution and sulfuric acid (H_2_SO_4_), known as the curing process, aiming to begin the copper sulfation process in the oxidized minerals or sulfated minerals (cured with mixed sulfuric acid and chlorides solutions [44,45]). The mineral is discharged by means of a spreader machine, depositing it in a very organized manner and forming a continuous embankment from 6 to 8 m high: The leaching heap. Above this heap, a drip irrigation system is installed, and sprinklers cover all the exposed area. Under the heaps of materials to be leached, a waterproof membrane is installed in order to provide a system of drains (grooved pipes) that allow to collect the Pregnant Leach Solution (PLS) that can infiltrate through the heap [46].

**Figure 1:**
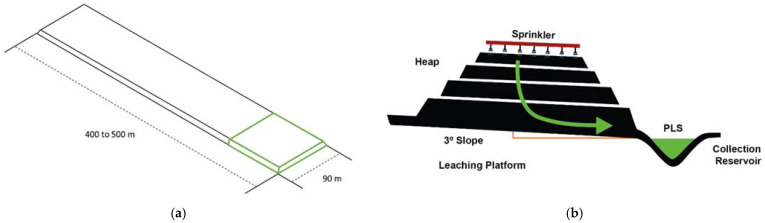
Dimensions (**a**) and cross section (**b**) of a conventional leaching heap.

Then, depending on the characteristics (physical and/or chemical) of the mineral, commercial percolation leaching can be grouped into the following categories [47]: in situ leaching (ISL), (underground), dump leach (DL), (mined (uncrushed) mineral), heap leach (HP, see Figure 1) (crushed and/or agglomerated mineral); vat leach (VL) (crushed mineral or concentrates); and agglomerated fines heap leach (AFHL) (crushed mineral or concentrates).

### 2.2. Bibliometric Analysis

**Figure 2:**
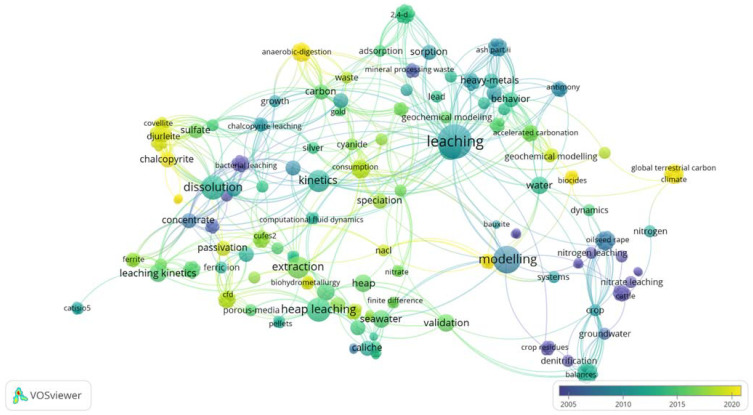
Networks in keywords of bibliography consulted (VOSviewer Software, version 1.6.17).

The bibliometric analysis indicates that from the information available about mineral leaching in the reference base “Web of Science”, there is a meaningful relationship between modeling, leaching, heap leaching, kinetics and dissolution. Nevertheless, there are no evident relationships with modeling based on machine learning, which does not indicate that there are no documents in this regard, rather the number of target documents is not substantial. Network visualization indicates the existence of different centered clusters during the leaching process, the kinetics process and the mineralogy process (see Figure 2), along with the time variation of the field of study during recent decades, underscoring the study of sulfur minerals, such as chalcopyrite and covellite, anaerobic digestion (associate to bioleaching processes) and the use of water or themed climates.

## 3. Mineral Leaching Modeling

Many leaching models were developed in the literature, contributing to mathematically expressing the phenomena that occur during the leaching process of metal minerals, with the purpose of studying the extraction kinetics and preparing models available to simulate or optimize the metallic values. The leaching process was modeled by many authors, using a wide variety of techniques, even inside the category of mathematical modeling, which is checked later as a sequence of time. Heap leaching is the most relevant industrial application of this discipline because it is an easy way to extract valuable metals from low grade minerals. However, the design of the leaching modeling dynamic is far from being adequate. Consequently, it is necessary to develop models that allow to predict recoveries as exact as possible, models which Barlett [48] define as two types: empirical and deterministic. The empirical data is based on historical data, but it requires a considerable amount of data to parameterize a heap, and considering the number of variables involved in leaching operations, the expansion of the models to bigger heaps is unfeasible, and the installation costs are still considerably high [49].

In modeling from the leaching process dynamic, it is considered that a chemical reaction takes place between the liquid reagent *A* and the solid reagent *B* according to Equation (1), where *b* is the stoichiometric number. Reagent *A* breaks through to the minerals with an effective diffusivity (*D_e_*). It is supposed that *B* homogeneously diffuses all over the particle or mineral cluster, this way all the solid zone or stalled can be modeled as just one reagent. The reactive, as well as the reagent, concentrations, *C_A_* and *C_B_*, possess units of molar quantity per mineral volume unit.

(1) A+bB→Products

Due to the lack of empirical models applied at industrial scales, models based on deterministic methods are developed (mainly) to represent the dynamic of the heap leaching process. The deterministic models are created using the dominant physio-chemical factors; nevertheless, due to the complexity of the process, modeling becomes challenging since it includes important processes such as the transport of fluids through particles and the litter heap, reactions such as the dissolution of metals and some other species that take place at the surfaces of mineral grains, and some arising complications derived from the effect of additional interactions such as biological activity processes, the movement of gases or heat transport.

### 3.1. Generic Mineral Leaching Modeling

Within the models developed to represent the reaction dynamics of mineral particles exposed to solvents, the first and best-known models are the shrinking core model (SCM) and progressive conversion models (PCM).

The SCM model was first developed by Yagi and Kunii [50], which includes three mechanisms that determine the overall conversion rate of the solid reagent: (i) mass transfer from the environmental fluid through the fluid layer that surrounds the particle, (ii) dissemination through the inert product layer that remains when the core contracts, and (iii) chemical reaction on the core surface. This model is appropriate when there is a huge reaction zone. The solid reagent distributed all over the particle gradually runs out according to the concentration of local reagent fluid. After, the SCM reaction zone is considered thin, disappearing in some dimensionless core radius $\xi =r_{c}/R$. In addition, the *X* extractions, or the stage of conversion per volume unit can be calculated by ξ, for example, the following equation: $X=1-\xi^{3}$ [50]. The general form of the SCM model is provided by the solution of the diffusion–reaction equation in spheric coordinates (see Equation (2)), while the limit conditions for spheric particles are: in the solid particle surface (see Equation (3)) and the mobile interface, $r_{c}$ (see Equations (4) and (5)), where $C_{A}$ is the concentration of component *A*, $C_{Ao}$, $C_{As}$ and $C_{Ac}$ are the reagent fluid concentrations *A* for the most part of the fluid stream, in the particle surface and the core surface, respectively. $k_{mA}$ is the mass transfer coefficient of component *A*, $C_{So}$ is solid reagent concentration in the unreacted core, assumed constant for uniform particles, and ϵ is the void porous layer of the particle.

(2) $$\epsilon \frac{\partial C_{A}}{\partial t}=D_{eA}\left[\frac{\partial^{2}C_{A}}{\partial r^{2}}+\frac{2}{r}\frac{\partial C_{A}}{\partial r}\right]\;|\;R\leq r\leq r_{c}$$

(3) $$D_{eA}{\left(\frac{\partial C_{A}}{\partial r}\right)}_{R}=k_{mA}\left(C_{Ao}-C_{As}\right)$$

(4) $$D_{eA}{\left(\frac{\partial C_{A}}{\partial r}\right)}_{r_{c}}=\alpha k_{s}C_{So}C_{Ac}$$

(5) $$-D_{eA}{\left(\frac{\partial C_{A}}{\partial r}\right)}_{r_{c}}=\alpha C_{So}\left(\frac{dr_{c}}{dt}\right)$$

The SCM model’s assumptions may not match accurately with the reality, an issue that is considered by Wen [51] and Ishida et al. [52], in which no simple catalytic cases are analyzed between solids and fluids, and the considerations expand to formulate a general model that can be applied to a wide variety of situations. The PCM model considers that the modeling of the particle leaching process must take into account that the solid contains enough vacuum so that the fluid reagent can diffuse inside the solid. Wen [51] contemplates that the reaction between the fluids and the solid is produced homogeneously over the solid, producing a gradual variation in the concentration of the solid reactant inside the particle, for which the homogeneous model is generated for the analysis of solid–fluid reactions in porous environments with large effective diffusivities and invariants during the reaction. The homogeneous model is not completely accurate, since most cases of solid–fluid reactions can probably be described by an intermediate model. Considering the type of solid–fluid reactions represented by Equation (1), since the reaction is faster near the surface than inside the particle, after a certain time, the solid reagent near the surface will be completely exhausted, forming an inert product, an ash layer [51]. The reaction period before the formation of the ash layer is designated as the first stage and the period following the formation of the ash layer is the second stage. The material balance for fluid reagent A and reagent S for spherical particles under a pseudo-steady state is shown in Equations (6) and (7).

(6) $$0=D_{eA}^{\prime }\left[\frac{\partial^{2}C_{A}^{\prime }}{\partial r^{2}}+\frac{2}{r}\frac{\partial C_{A}^{\prime }}{\partial r}\right]-\alpha k_{v}C_{So}C_{A}^{\prime }\;\;\frac{\partial C_{S}}{\partial t}=-k_{v}C_{So}C_{A}^{\prime }$$

(7) $$D_{eA}^{\prime }\frac{\partial C_{A}^{\prime }}{\partial r}=k_{mA}\left(C_{Ao}-C_{A}^{\prime }\right)\;\mathrm{At}\;r=R\;\frac{dC_{A}^{\prime }}{dr}=0\;\mathrm{At}\;r=0\;C_{S}=C_{So}\;\mathrm{At}\;t=0$$

Here the reaction rate is considered first-order according to fluid reagent A but independent of the concentration of the solid reagent. The homogenous model developed by Wen [51] is provided in Equation (8), while the conversion rate per volume unit X* is given by Equation (9).

(8) $$C_{A}=0\;\mathrm{para}\;0\leq r\leq r_{m}\;\frac{C_{A}}{C_{Ao}}=\frac{1-\xi_{m}/\xi }{1-\xi_{m}+\xi_{m}/N_{Sh}}\;\mathrm{para}\;r_{m}\leq r\leq R\;\;X=1-\xi_{m}{}^{3}$$

(9) $$X*=1-3{\left(1-X\right)}^{2/3}+2\left(1-X\right)$$

Analyzing the process from a systematic point of view, mineral leaching processes, in general, can be described as two types of models: a micro and a macro model. The micro model deals with the changes in the system [53] through a mathematical equation for single-particle leaching (variants of the SCM model), which is integrated over the residence time distribution applicable to the leaching system. In the macro model, on the other hand, Peters [53] uses material and heat balances, employing surface integrals, thus obtaining a general model describing batch (see Equation (10)) and continuous (see Equation (11)) leaching in agitated leach tanks, where *P*(*t*) is the residence time distribution, $\Psi \left(r_{0}\right)$ is the particle size distribution and ${\left[\frac{A_{t}}{W_{0}}\right]}_{r_{0}}$ is a function representing the leached area per mineral weight at the particle level.

(10) $${\left(\frac{A}{W_{0}}\right)}_{t}=\int_{r_{0}\left(min\right)}^{r_{0}\left(max\right)}{\left[\frac{A_{t}}{W_{0}}\right]}_{r_{0}}\Psi \left(r_{0}\right)dr_{0}$$

(11) $${\left(\frac{A}{W_{0}}\right)}_{t}=\int_{r_{0}\left(min\right)}^{r_{0}\left(max\right)}\int_{0}^{t\left(max\right)}{\left[\frac{A_{t}}{W_{0}}\right]}_{r_{0}}\Psi \left(r_{0}\right)P\left(t\right)dtdr_{0}$$

Dixon and Hendrix [54,55] proposed another approach considering that the leaching phenomenon occurs at different size and time scales and that different phenomena particles in the leaching process, deriving from a mathematical model in dimensionless form for the heap leaching of one or more solid reagents, form spherical, porous and non-reactive mineral particles [54] and a general unsteady-state model for the leaching of one or more reagent species [55]. The mathematical formulation of Dixon and Hendrix [54] assumes the existence of *n* solid reagents, $B_{i}$, which are dissolved by a single reagent A (see Equation (12)). Then, assuming that the dissolution of each solid reagent is of the first-order in the concentration of a rate control reagent and varying the order in its own solid concentration, the dissolution rate of solid reagent *i* is expressed as shown in Equation (13), where $C_{pi}$ is the solid concentration of reagent *i* at the pore walls at particle radius *r*, $k_{pi}$ is the reaction rate constant expressed per unit particle mass, $C_{A}$ is the concentration of reagent at particle radius *r* and $\phi_{pi}$ is the reaction order at the solid concentration of solid reagent *i*. Since each reaction within the particle involves the consumption of reagent *A*, the mass balance of reagent *A* within the porous sphere takes the form of a continuity equation with a summed consumption term, as shown in Equation (14), where $D_{Ae}$ is the effective diffusivity of reagent *A* within the pores of the particles, $\rho_{0}$ is the specific gravity of the mineral matrix, $\varepsilon_{o}$ is the porosity of the particle and $C_{Ab}$ represents the concentration of reagent *A* in external solution to the particle.

(12) $$A+\overset{n}{\underset{i=1}{\sum }}b_{i}B_{i}\to dissolved\;products$$

(13) $$\frac{dC_{pi}}{dt}=-k_{pi}C_{pi}^{\phi_{pi}}C_{A};\;C_{pi}\left(r,0\right)=C_{pi0}$$

(14) $$\varepsilon_{o}\frac{\partial C_{A}}{\partial t}=D_{Ae}\left[\frac{\partial^{2}C_{A}}{\partial r^{2}}+\frac{2}{r}\frac{\partial C_{A}}{\partial r}\right]-\rho_{0}\left(1-\varepsilon_{o}\right)\overset{n}{\underset{i=1}{\sum }}\frac{k_{pi}C_{pi}^{\phi_{pi}}C_{A}}{b_{i}}\;C_{A}\left(r,0\right)=0;\;C_{A}\left(R,t\right)=C_{Ab};\;\frac{\partial C_{A}}{\partial r}\left(0,t\right)=0$$

In contrast to the assumption that the leaching of mineral deposits at the particle surface and leaching in the pore deposits are serially occurring dissolution processes, Dixon and Hendrix [54] assume that these two processes occur in parallel and keep the assumptions of the intra-particle reaction order, resulting in the dissolution rate of the solute reagent i at the particle surface given by Equation (15), where $C_{si}$ is given as the solid concentration of solute reagent i at the particle surface and $k_{si}$ is the rate of reaction speed. For the purpose of finding the important parameters of the model, Dixon and Hendrix [54] define a set of dimensionless variables (see Equation (16)) where $C_{A0}$ is a reference reagent concentration, whereas the restructuring in dimensionless terms, is represented in Equations (17)–(20).

(15) $$\frac{dC_{si}}{dt}=-\frac{3k_{si}C_{si}^{\phi_{si}}C_{Ab}}{R\rho_{0}\left(1-\varepsilon_{0}\right)};\;C_{si}\left(0\right)=C_{si0}$$

(16) $$\alpha =\frac{C_{A}}{C_{A0}};\;\alpha_{b}=\frac{C_{Ab}}{C_{A0}};\;\sigma_{pi}=\frac{C_{pi}}{C_{pi0}};\;\sigma_{si}=\frac{C_{si}}{C_{si0}};\;\xi =\frac{r}{R};\;\tau =\frac{D_{Ae}t}{\varepsilon_{0}R^{2}}$$

(17) $$\beta_{i}=\frac{\varepsilon_{0}b_{i}C_{A0}}{\rho_{0}\left(1-\varepsilon_{0}\right)C_{Ei0}}$$

(18) $$\lambda_{i}=\frac{C_{si0}}{C_{Ei0}}$$

(19) $$\kappa_{pi}=\frac{\rho_{0}\left(1-\varepsilon_{0}\right)k_{pi}C_{pi}^{\phi_{pi}}R^{2}}{b_{i}D_{Ae}}$$

(20) $$\kappa_{pi}=\frac{3k_{si}C_{si}^{\phi_{si}}R}{b_{i}D_{Ae}}$$

The continuity equation for reagent *A* is provided in Equation (21), while the dissolution rate of the solid reagent i at the particle surface is given in Equation (22).

(21) $$\frac{\partial^{2}\alpha }{\partial \xi^{2}}+\frac{2}{\xi }\frac{\partial \alpha }{\partial \xi }-\overset{n}{\underset{i=1}{\sum }}\kappa_{pi}\sigma_{pi}^{\phi_{pi}}\alpha =\frac{\partial \alpha }{\partial \tau }\;s.a.\;\alpha \left(\xi ,0\right)=0;\;\alpha \left(1,\tau \right)=\alpha_{b};\;\frac{\partial \alpha }{\partial \xi }\left(0,\tau \right)=0$$

(22) $$\frac{d\sigma_{si}}{d\tau }=-\frac{k_{si}\beta_{i}}{\lambda_{i}}\sigma_{si}^{\phi_{si}}\alpha_{b}\;s.a.\;\sigma_{si}\left(0\right)=1$$

Next, Dixon and Hendrix [54] define functions that include the fractional conversion of the solid reagent $X_{i}$ (fraction of extractable solid reagent that has been dissolved) and the effectiveness factor $\eta_{i}$ (see Equation (23)), while the governing equations are provided in Equations (24)–(26). Fractional, homogeneous or progressive conversion considers the whole particle to be the reaction zone and generally, a shrinking core radius is not modeled. *S* becomes a prognostic variable that is attached to *C* through a source term added to Equation (23). Whereas most mineral grains can be found lining the pores of mineral particles, a proportion λ may reside on the exterior surface; therefore, in addition to determining a concentration of porous solids $\sigma_{pi}$ within the particle (or equivalently, within the particle group), we also determine a concentration of solids on the surface $\sigma_{si}$.

(23) $$X_{i}=3\left(1-\lambda_{i}\right)\int_{0}^{1}\left(1-\sigma_{pi}\right)\xi^{2}d\xi +\lambda_{i}\left(1-\sigma_{si}\right)$$

(24) $$\frac{\partial C_{i}}{\partial t*}=\frac{1}{\xi^{2}}\frac{\partial }{\partial \xi }\left(\xi^{2}\frac{\partial C_{i}}{\partial \xi }\right)-\kappa_{pi}\sigma_{pi}^{\phi_{pi}}\alpha$$

(25) $$\frac{d\sigma_{pi}}{dt*}=-\frac{\beta_{i}\kappa_{pi}}{1-\lambda_{i}}\sigma_{pi}^{\phi_{pi}}\alpha$$

(26) $$\frac{d\sigma_{si}}{dt*}=-\frac{\beta_{i}\kappa_{si}}{\lambda_{i}}\sigma_{si}^{\phi_{si}}\alpha_{b}$$

where the values are assumed from 0, the beginning of the leaching cycle, to 1, at the end of the leaching cycle, while the above model is summed up to the standard PCM when $\lambda_{i}=0$. In addition, Dixon and Hendrix [56] show that it is possible to represent different distributions of mineral grains by fitting $m_{p}$ or $m_{s}$. The classical shrinking core model is recovered when m=2/3, and wider log-normal distributions can be approximated by choosing higher values. Subsequently, Dixon [57] develops a new method for modeling multiparticle leaching kinetics in multistage continuous reactors by calculating the fraction of unreacted leachable solids using a multiple convolution integral dependent on the residence time distribution function E(θ) and the particle size distribution $f\left(\xi_{0}\right)$ (see Equation (27)), and then solving it numerically using Gaussian quadrature.

(27) $$1-{\overset{=}{X}}_{B}=\int_{0}^{\infty }\int_{\xi_{0}\left(X_{B}=1\right)}^{\xi_{0}^{max}}\left(1-X_{B}\right)f\left(\xi_{0}\right)d\xi_{0}E\left(\theta \right)d\theta$$

Erikson and Destouni [58] use a probabilistic Lagrangian approach to model reagent underground transport to study various processes that may affect long-term field-scale copper leaching from waste rock piles by modeling the concentration of transient mass balance equation (see Equation (28)) for copper along with an arbitrary streamline in the flow field through waste rock piles.

(28) $$\frac{\partial c}{\partial t}+v\frac{\partial c}{\partial z}=-\frac{1}{\theta }\frac{\partial c^{*}}{\partial t}\;|\;\frac{\partial c^{*}}{\partial t}=-kc^{*};\;c^{*}\left(t\right)=c_{0}^{*}e^{-kt}$$

Considering the above, for no catalytic reaction of mineral particles with a surrounding fluid, Levenspiel [59] mainly presents two simple idealized models, the progressive conversion model (PCM) and the shrinking core model (SCM). In the SCM the reactions first occur in the outer skin of the particle. The reaction zone moves towards the solid, leaving behind all the deedless solid converted material. Therefore, at any time, there is an unreacted material core which shrinks in size during the reaction, whereas in the PCM model, the fluid comes in and always reacts through the particle, probably at different speeds in different places inside the particle. Consequently, the solid reagent converts continuously and progressively.

Bouffard and Dixon [60], on the other hand, model hydrodynamic properties for the heap leaching process, such as mixed lateral pore diffusion (MSPD, see Equation (29)) and profile lateral pore diffusion (PSPD, see Equation (30)) with uniform or distributed pore lengths, in order to simulate solute transport through the flow channels and stagnant pores of an unsaturated heap. In addition, the mass balance in the liquid phase is described for pores of uniform length *X* (see Equation (31)), and for pores of different lengths, defining a dimensionless pore length (Ξ), which normalizes the pore length X to a reference pore length X*. The normalized distribution is provided in Equation (32).

(29) $$\frac{\partial C_{s}}{\partial t}=\frac{1}{\varepsilon_{s}}ka_{v}\left(C_{f}-C_{s}\right)\;|\;C_{s}\left(0\right)=0$$

(30) $$\varepsilon_{s}\frac{\partial C_{s}}{\partial t}=D\left(\frac{\partial^{2}C_{s}}{\partial x^{2}}+\frac{n}{x}\frac{\partial C_{s}}{\partial x}\right)\;|\;C_{s}\left(x,0\right)=0$$

(31) $$\varepsilon_{f}\frac{\partial C_{f}}{\partial t}=-\mu \frac{\partial C_{f}}{\partial z}-\frac{D\left(n+1\right)}{X}\frac{\partial C_{s}}{\partial x}$$

(32) $$\overset{\Xi_{max}}{\underset{\Xi_{min}}{\int }}n\left(\Xi \right)d\Xi =\overset{1}{\underset{0}{\int }}m\Xi^{m-1}d\Xi =1$$

The work developed by Bouffard and Dixon [60] concluded that of the factors tested, flow rate and height were the most significant.

Some criteria that determine the issues associated with the SCM model in liquid–solid systems in hydrometallurgical processes are shown in Liddell [61], fitting various models as a function of a geometric particle. The models of Equations (33)–(36) were fitted for spherical particles under control reaction, spherical particles under product layer diffusion control, cylindrical particles under reaction control and cylindrical particles under product layer diffusion controls, respectively. The additional kinetics models suggested in the literature for modeling the leaching process are shown in Table 2.

(33) $$t\propto 1-{\left(1-X\right)}^{1/3}$$

(34) $$t\propto 1-3{\left(1-X\right)}^{2/3}+2\left(1-X\right)$$

(35) $$t\propto 1-{\left(1-X\right)}^{1/2}$$

(36) t∝X+(1−X)ln(1−X)

On the other hand, in McBride et al. [70], the computational model of variable saturated flow in porous media in complex three-dimensional geometries, such as a leach pad, is presented using CFD based on existing conventional finite volumes, targeting its functionality to modelling of a complex fluid suite interaction and thermal and chemical reaction process physics. Flow through variably saturated porous media is characterized by the classical Richards equation combined with one of several laws to relate the pressure head to the moisture content of the porous medium. There are three standard forms of the Richards equations: based on *h* (pressure head), based on θ (moisture content) and a “mixed” form where both variables are used, as shown in the Equations (47)–(49), respectively. McBride et al. [70] conclude that the CFD-based model flow algorithm shows the convergent behavior of the transformed equations, allowing solutions on a much coarser mesh and employing larger time steps, and is comprehensive in the sense that the potential of the tool as a basis for industrial heap leach pad modeling is demonstrated in a basic three-dimensional geometry.

(47) $$C\left(h\right)\frac{\partial h}{\partial t}=\nabla \left[K\left(h\right)\nabla h\right]+\frac{\partial K\left(h\right)}{\partial z};\;C\left(h\right)=\frac{\partial \theta }{\partial h}$$

(48) $$\frac{\partial \theta }{\partial h}=\nabla \left[D\left(\theta \right)\nabla \theta \right]+\frac{\partial K\left(\theta \right)}{\partial z}$$

(49) $$\frac{\partial \theta }{\partial t}=\nabla \left[K\left(h\right)\nabla h\right]+\frac{\partial K\left(h\right)}{\partial z}$$

Subsequently, in McBride et al. [71] a complete mathematical model of mineral leaching at multiple scales is presented, capturing details of reactions at a particle level and complete transport problems at the scale of the entire heap. The host code PHYSICA, provides a modular three-dimensional finite-volume unstructured mesh framework for multiphysics modeling by solving a general conservation equation (see Equation (50)), while the flow through porous media is characterized by means of the Richards equation. The continuity equation for the disperse-convective transport of multiple solutes in porous media used by McBride et al. [72], and described above by Bear [73], is shown in Equation (51).

(50) $$\frac{\partial \left(T_{\varphi }\varphi \right)}{\partial t}+div\left(C_{\varphi }\underline{\mu }\varphi \right)=div\left(D_{\varphi }grad\left(\varphi \right)\right)+S_{\varphi }$$

(51) $$\frac{\partial \left(\theta C^{i}\right)}{\partial t}-\nabla \left(\theta D\cdot \nabla C^{i}\right)+\nabla \left(qC^{i}\right)=S^{i}$$

In a future work, McBride et al. [74] developed a comprehensive heap leach model within a CFD framework, providing a modeling tool that captures the reagent dissolution of low grade oxide and sulfide minerals. The simulated and optimized CFD-based models involve complex reaction sets [72,75], and an optimization tool was incorporated into the model to allow the automated search of multiple parameter values with the aim of both improving the fit and simulating large-scale forecasts [74]. The model was then calibrated to model the recovery of different minerals, such as copper, gold and silver.

Finally, another interesting study in mineral leaching modeling was developed by Meirmanov et al. [76], where several processes related to leaching dynamics were analyzed by applying a general mathematical approach, based on a detailed consideration of mechanics and chemistry laws at the pore scale, modeling the process at the microscopic and macroscopic scales. The microscopic model uses a continuity equation for a generalized motion of continuous media, while in the macroscopic model, micro models are scaled to analyze the processes realistically.

### 3.2. Copper Leaching Modeling

The first analytical model was described by Taylor and Wellan [77], where the mineral recovery was considered as the inverse exponential function (see Equations (52) and (53), where k and c are constant terms), whereas the formulation of the shrinking core model (SCM)–speed of limited reaction by diffusion indicates that partially leached cores show a sharp boundary between the unreacted inner core and the leached shell, which is analytically formalized in Equation (54), in which the change in contracted particle core is described, and the recovery equation, according to the size of the contracted core [78], is presented, where $\;r_{c}\;$ represents the unreacted core radius, R is particle radius, α is grams of consumed acid per gram of leached copper, ρ is solid density, σ is particle surface area, D is effective diffusivity, V is volume factor and $C^{0}$ is solution initial concentration.

(52) $$Mineral\;recovery=\left(1-e^{-k\left(t+c\right)}\right)$$

(53) $$Mineral\;recovery\;\left(\%\right)=100\left(1.0-{\left(\frac{r_{c}}{R}\right)}^{g}\right)$$

(54) $$-\rho \alpha r_{c}^{2}\left(\frac{1}{r_{c}}-\frac{1}{R}\right)dr_{c}=\frac{\sigma D}{v}C^{0}dt$$

Looking back to Taylor and Whelan modeling [77], Botz and Marsden [79] use the Equation (52) model to predict the copper production of industrial heap leaching operations.

Subsequently, the standard SCM was modified to capture phenomena that are characteristic of the leaching process [78,80]. Braun et al. [80] observed increases in leaching rates in the later stages of their experiments, attributing them to the generation of cracks and fissures, particularly in large mineral particles. Braun et al. [80] present the reaction rate (dn/dt) enhanced through a geometrical particle shape factor $\phi_{io}$, which effectively scales the particle radius and the particle size, or in other words, the leachable outer of the particle (see Equation (55)). In contrast, Roman et al. [78,80] proved the effectiveness of the model presented in Equation (54), where the unreacted core radius differential ($r_{c}$) depends mainly on acid concentration in the system (see Equation (56)). Another application of the SCM model was developed by Koninshi et al. [81], where copper leaching rates from natural covellite particles were studied.

(55) $${\left(\frac{dn}{dt}\right)}_{i}\propto \left(\frac{4\pi r_{i}^{2}}{\phi_{io}}\right)$$

(56) $$-\rho \alpha r_{c}^{2}\left(\frac{1}{r_{c}}-\frac{1}{R}\right)dr_{c}=\frac{\sigma D}{v}C^{0}dt$$

Quast [82] modeled the leaching process setting up the Mclaughlin and Agar model [83] in a copper mine in an Atacamite form in order to study the suitability of the material for agitation leaching processing. De Andrade Lima [84], on the other hand, simulates the transient evolution of the dissolved chemical species in leaching processes by modeling the recovery of solid reagent $\alpha_{B}$ (see Equation (57)) following an ordinary differential equation [85]. De Andrade Lima [84] assumes that the solution flow in the solid bed is unidirectional without dispersion and the solid–fluid reaction is described by a diffusion control model that is analytically integrated for each time step, whereas the set of models includes variables such as content of leachable chemical species, flow rate and concentration of the leached agent, particle size and residence time of the solution in the bed, among others. The sensitivity analysis shows that the apparent diffusivity of the leaching agent in the solid particles and the average residence time of the solution in the bed are parameters that strongly impact the simulation results.

(57) dαBdt≅(3lcCACBo)(R2DAe)[(1−αB)−13−1]+(Rlc kS CBo)(1−αB)−23+RklsAls

Sheikhzadeh et al. [86] model in aggregate form the unsaturated liquid flow in the bed uniform spherical mineral particles, developing an unsteady two dimensional model based on mass conservation equations in both the liquid phase in the bed and in the particles. The mass conservation equation of the unsaturated liquid flow through the porous bed is presented in Equation (58), while the diffusion of the liquid into the particle is presented in Equation (59). Fluctuations in the degree of saturation depend not only on the period, but also vary with the intrinsic permeability; the depth of the bed decreases as the intrinsic permeability or depth increases. The saturation degree increases as the infiltration period increases.

(58) $$\frac{\partial \left(\varepsilon_{l}\rho_{l}\right)}{\partial t}=-div\left(\rho_{l}u\right)+q_{l}\;\left|\;u=-K_{l}grad\left(\Phi \right)\;\right|\;\Phi =h_{l}-z$$

(59) $$\frac{\partial S_{p}}{\partial t}=\frac{1}{r^{2}}\frac{\partial }{\partial r}\left(r^{2}D_{p}\frac{\partial S_{p}}{\partial r}\right)+\frac{q_{lp}}{\varepsilon_{p}\rho_{l}}$$

In the same line as Liddell [61], Razavizadeh and Afshar [87] model the surface reaction conversion function for copper oxides in two stages. The kinetic study showed that the dissolution for stage 1 was a diffusion-controlled reaction, and the dissolution of stage 2 was a chemical-controlled reaction. About 85% of malachite dissolution (the copper oxide studied) occurred in stage 1, while 15% occurred in stage 2. In addition to the study of malachite leaching, Yaras and Arslanoglu [88] investigate the copper leaching kinetics of malachite ore using formic acid as an organic leaching reagent. The kinetic model was used to indicate the effects of these parameters on copper leaching from malachite ore in formic acid solution, considering particle size, acid concentration, leaching time, formic acid/malachite ratio, reaction temperature and stirring rate as variables.

On the other hand, returning to analytical modeling, Mellado et al. [89] present analytical models describing heap leaching, based on the Bernoulli equation and using constitutive equations for different variables, which in simple form can be applied to analytical, optimization and scaling design issues. The final models developed by Mellado et al. [89] consider two size scales (both at the particle and heap level) and are presented in Equation (60), where variants are generated to incorporate and study the effect of heap height, particle size and the superficial velocity of lixiviant flow through the bed. Continuing with this analysis framework, Mellado et al. [90] present analytical models (based on first-order ordinary differential equations) to scale up the heap leaching process of solid reagents from porous pellets (model used later to design, plan and optimize a heap leach system [91]). In the process of scaling up the models developed above [89], the authors worked under the assumptions that the full recovery of every particle is not possible, since it never occurs in practice, since in the heap, things do not behave as ideally as the phenomenological models assume, due to the existence of gutters, clusters and particles that hinder diffusion processes. Infinite time recovery ($E^{\infty }$) is defined and modeled by Mellado et al. [90], while the aggregate recovery model considers variables such as heap height (Z), superficial velocity of leaching flow ($u_{s}$), volumetric fraction of the bulk solution in the bed ($\epsilon_{b}$), effective diffusivity of the solute within the particle pores ($D_{Ae}$), particle radius (R) and particle porosity ($\epsilon_{b}$). 

The simplicity of the models developed by Mellado et al. [89,90] allows to overcome the mathematical complexities of models based on partial differential equations and the characteristic issue of the empirical models. Continuing with analytical modeling and its validation, Mellado et al. [92] apply sensitivity analysis to validate an analytical leaching model, concluding that a model based on a combination of two-level (particle and heap) leaching kinetics is adequate to represent the heap leaching process of solid reagents from porous granules. Finally, in Mellado et al. [93] analytical models are presented and it is shown how the uncertainty in the independent variables and the process parameters impact the response. In comparison, in Mellado et al. [94], a posteriori analysis of analytical models of the heap leach phase using uncertainty and sensitivity analysis is developed.

(60) $$E\left(t\right)=E^{\infty }\left(\alpha ,\beta ,\gamma ,Z\right)\left[1-\lambda e^{-k_{\theta }\left(\frac{u_{s}}{\epsilon_{b}Z}t-\omega \right)}-\left(1-\lambda \right)e^{-k_{\tau }\frac{D_{Ae}}{R^{2}\epsilon_{0}}\left(t-\frac{\epsilon_{b}Z}{u_{s}}\omega \right)}\right]\;E^{\infty }\left(\alpha ,\beta ,\gamma ,Z\right)=\frac{\alpha }{Z^{\gamma }+\beta }\left[1-{\left(1-\frac{Z^{\gamma }+\beta }{\alpha }\right)}^{Z/2R}\right]\;\underset{Z\gg R}{\lim}E^{\infty }\left(\alpha ,\beta ,\gamma ,Z\right)=\frac{\alpha }{Z^{\gamma }+\beta }$$

Naderi et al. [95], on the other hand, study the chemical leaching kinetics of chalcopyrite from low-grade copper ore using the SCM model, modeling the leaching kinetics as a weighted product of the following steps: the diffusion of the leachant through the liquid film surrounding the particle; the diffusion of the leachant through the product layer at the surface of the unreacted core; the chemical reaction of the leachant at the surface of the core with reactant. In this approach, for finding the controlling steps in the leaching process, the simultaneous actions of these steps, which act in series, are combined as showed in Equation (61), where the contribution of each step can be revealed by fitting the experimental data to Equation (61) and evaluating the constant parameters ($\tau_{F},\;\tau_{P},\;\mathrm{and}\;\tau_{R}$).

(61) $$t=\tau_{F}X+\tau_{P}\left[1-3{\left(1-X\right)}^{2/3}+2\left(1-X\right)\right]+\tau_{R}\left[1-{\left(1-X\right)}^{1/3}\right]$$

Similar to the model fitted by Mellado et al. [89], and in a more simplified form, Ekmekyapar et al. [96] investigate the kinetics of copper cementation using a rotating aluminum disc from leaching solutions containing copper ions. The kinetic analysis was performed according to first-order kinetics (see Equation (52)), and it was found that the cementation rate was diffusion controlled. Similarly, Marsden and Botz [97] model the extraction of metal (including copper) following first-order extraction curves, considering that the behavior of the heap could be modeled using a system of first-order equations [89].

Cariaga et al. [98] developed and implemented a two-dimensional mathematical model to describe the leaching of copper mineral tailings using H_2_SO_4_ as leaching agent. The mathematical model consists of a system of differential equations: two diffusion–convection–reaction equations with Neumann boundary conditions, and an ordinary differential equation (see Equation (62)) where $u_{1}$, $u_{2}$ and $u_{3}$ are the H_2_SO_4_ concentration and copper concentration in liquid and solid phase, respectively, *D* is the diffusion–dispersion tensor and the vector *V* is the fluid flow velocity. The system is complemented with non-homogeneous flow contour conditions, which correspond to the physical behavior of the irrigation and infiltration processes in leaching piles. The system of heap leach transport equations used is very similar to that of Cariaga et al. [99] which are derived from the compositional flow model considered by Kacur and Van Keer [100]. The results of the model generated by Cariaga et al. [98] show that the model satisfactorily predicts that main trends exhibited by the phenomenon studied, i.e. the time evolution of acid and copper concentration in the liquid solution extracted from the tailings.

(62) $$\partial_{t}u_{1}+\nabla \cdot \left(\alpha_{1}\left(u_{1}\right)v-D\nabla \alpha_{1}\left(u_{1}\right)\right)=\Phi_{1}\left(u_{1}\right)\;\partial_{t}u_{2}+\nabla \cdot \left(\alpha_{2}\left(u_{2}\right)v-D\nabla \alpha_{2}\left(u_{2}\right)\right)=\Phi_{2}\left(u_{1},u_{3}\right)\;\partial_{t}\left(\Psi \left(u_{2}\right)+u_{3}\right)=\Phi_{3}\left(u_{1},u_{3}\right)$$

Simplifications of the fits models presented by Mellado et al. [89,90,93,94] are developed in Saldaña et al. [101], where analytical models (Equation (60)’s variants) are fitted for the extraction of copper from oxidized copper minerals, leached only in acid media, and sulfide copper minerals (secondary sulfides), leached in acid media with the addition of chloride [21,22,23,102] (at different concentrations), to subsequently determine the impact of the change in the operation modes, obtaining a discrete event simulation framework.

The basic formulation of a comprehensive heap leaching copper model based on CFD technology, together with its parameterization and validation against laboratory column test data is developed by Bennett et al. [75]. The modeling of the copper sulfide heap leach system developed considers: the modeling of liquid phase transport (via the Darcy flow shown in Equation (63)), hydraulic conductivity (described by the van Genuchten equation, Equation (64) [103]), transport in the gas phase (basic continuity equation for the gas phase in Equation (65)) and mineral dissolution rate (see Equation (66)) [104]. Simulation results of the model developed by Bennett et al. [75] show that for both the small column and the large column, the modeling has a good fit, despite evidence that the behavior of both systems is different. The small particle size distribution in the small column leads to faster reactions than in the large column, which, in turn, leads to acid depletion and the precipitation of ferric salts, something that does not happen with slower reacting large particles in the large column.

(63) $$q=-K\left(\theta \right)-K\left(\theta \right)\left(-\frac{\partial \psi_{m}}{\partial \theta }\right)\frac{\partial \theta }{\partial z}$$

(64) $$K\left(\theta \right)=k_{s}S{\left(\theta \right)}^{0.5}{\left[1-{\left(1-S{\left(\theta \right)}^{1/m}\right)}^{m}\right]}^{2}$$

(65) $$div\left[\rho_{g}v_{g}\right]=S_{g}$$

(66) $$\frac{dr_{m}}{dt}=-\frac{3r_{m}}{4\pi r_{o}^{2}}\frac{M_{i}}{\rho_{ore}x_{i}}\frac{D_{eff}c_{o}A_{m}}{\left[3D_{eff}r_{o}c_{o}+2\left(r_{o}-r_{m}\right)r_{m}^{2}\left(1-\varepsilon_{p}\right)A_{m}\right]}$$

It should be noted that $A_{m}$ comes from the general expression of the kinetic rate equations, such as those produced by Paul et al. [105], which takes the general form $A_{m}=d\beta /dt=Ae^{-\beta /RT}$, with $\beta ={\left(r_{m}/r_{o}\right)}^{3}$ as the amount of reagent mineral.

A simpler modeling approach, in contrast with those presented above, is the one adjusted by Aguirre et al. [106], where an experimental design (based on response surface methodology, RSM) was developed, in which the central compound face approach [107] and quadratic model (regression model) were applied (see Equation (67)) to study the effect of independent variables such as temperature, ionic liquid concentration and chloride and H_2_SO_4_ on copper extraction from chalcopyrite (CuFeS_2_). Similarly, and previously, Liu et al. [108] optimized copper leaching from a low-grade flotation middling through RSM, studying the effect of key parameters, i.e., sulfuric acid concentration, nitric acid concentration and leaching time, on the leaching efficiency.

(67) $$Mineral\;recovery=\beta_{0}+\overset{n}{\underset{i=1}{\sum }}\beta_{i}x_{i}+\overset{n}{\underset{i=1}{\sum }}\overset{n}{\underset{j=1}{\sum }}\beta_{ij}x_{i}x_{j}$$

In Lin et al. [109], the apparent leaching kinetics of a particle within a heap leaching system is studied. The mathematical model implemented takes the form of partial differential equations set, describing the movement of the reagents, successively coupled to a mineral grain dissolution model, which is separated into the governing equation (see Equation (68)) and the boundary condition equations (see Equations (69) and (70)), whereas the assumptions of the SCM model are taken, relaxing the assumption of spherical particles and improving predictions by using the XMT image data of the internal structure of the particles. The main assumptions are mass transport in a quasi-steady state, surface kinetics are linear and uniform diffusivity. It was found out that these simulations can accurately predict both general leaching trends and the leaching behavior of mineral grains into classes according to their size and distance from the particle surface. Finally, the novelty introduced lies in the use of such particle-level technique to reduce the necessity of column experiments, concluding the apparent leaching kinetics depending on the distribution of mineral grains, in terms of size and position.

(68) ∇·(D∇C*)=0

(69) $$\nabla C*\cdot n{|}_{\partial MS}=-\frac{k_{react}}{D}C*$$

(70) $$\nabla C*\cdot n{|}_{\partial Rocks}=\frac{k_{react}}{D}\left(1-C*\right)\;|\;C*=\frac{C}{C_{ext}}$$

Although leaching models that are directly coupled to an increasingly smaller core model are successfully validated in small-scale experiments using an already provided mineral sample, they are hardly scalable or useful for generalization; therefore, choosing parameters that best fit laboratory results will be an iterative process if the particles follow a size distribution rather than having a uniform size [72,75,110]. Subsequently, a semi-empirical system that inherently captures particle heterogeneities is proposed in Ferrier et al. [111], ignoring the uniform size assumption. The model does not assume the geometry, physical structure or mineral grain distribution of the particles involved in the leaching process. The key assumption is that the effect of the current state of the mineral is mathematically separable from the conditions in which the mineral particles are exposed (chemical concentrations, temperature, pH, Eh, humidity, etc.). Similar models in the literature were proposed by Dixon and Hendrix [55] and extended by Ghorbani et al. [112]. Then, considering the cases where the reagent kinetics are nonlinear, Ferrier et al. [111] propose that it can be approximated by a modification of the SCM with linear kinetics, replacing the linear scale $C_{ext}$ (see Equation (71)) with a nonlinear scale (see Equation (72)), proposing a new semi-empirical approach where its make an even broader the conversion rate approximation and replacing the full partial differential equation with a product of functions (see Equation (73)), which reduced the computational cost and reduced the dependence on external reagent concentration, extending it to take into account other external conditions, such as temperature, pH, Eh and/or humidity.

(71) $$\frac{1}{\beta }\frac{d\xi_{c}}{dt*}=\frac{C_{ext}}{1/\kappa_{c}+\xi_{c}\left(1-\xi_{c}\right)}$$

(72) $$\frac{1}{\beta }\frac{d\xi_{c}}{dt*}=\frac{C_{ext}^{n\prime }}{1/\kappa_{c}+\xi_{c}\left(1-\xi_{c}\right)}$$

(73) $$\frac{dX}{dt}=k_{ext}\left(C_{ext\;1},C_{ext\;2},T_{ext},pH,Eh,\ldots \right)f\left(X\right)$$

On the other hand, Robertson [113] develops a one-dimensional model of solution flow and mineral leaching to demonstrate a dual-porosity approach whereby a mineral bed is divided into mobile (adjective) and stagnant (diffusion control) flow regimes. The solute balance is modeled using the standard advection–dispersion equation used in dispersion models; however, the term describing solute (copper) desorption from the solid to a liquid phase was replaced by a speed term from the SCM model. The advection–dispersion equation is now modified by replacing the sorption term, representing the change in mass of species, where $M_{Cumo}$ and $M_{Cuim}$ represent the copper mass per unit volume on contact with the mobile and immobile phases, as shown in the Equations (74) and (75), respectively.

(74) $$\frac{\partial \theta_{mo}c_{mo}}{\partial t}+\frac{\partial M_{Cumo}}{\partial t}=\frac{\partial }{\partial z}\left(\theta_{mo}D_{mo}\frac{\partial c_{mo}}{\partial z}\right)-\frac{\partial q_{mo}c_{mo}}{\partial z}-\omega_{mim}\left(c_{mo}-c_{im}\right)$$

(75) $$\frac{\partial \theta_{im}c_{im}}{\partial t}+\frac{\partial M_{Cuim}}{\partial t}=\omega_{mim}\left(C_{mo}-C_{im}\right)$$

Continuing with the application of the earlier developed models in McBride et al. [70], McBride et al. [114] use a robust CFD framework that incorporates techniques to account for local preferential flow paths in the heap leach system. The proposed new solute transport model includes a term that acts as a sink term $S_{i}^{cl}$ (see Equation (76)), which represents the liquid transfer rate from the matrix to the preferential flow channels, liquid that is channeled through preferential pathways without interaction with the mineral matrix. The channeled solution sink term depends on the local hydraulic properties of the medium and the saturation levels within the ore. Additionally, in McBride et al. [115], a three-dimensional heap is simulated under constant meteorological conditions, investigating continuous and intermittent irrigation, which shows that copper recovery per unit volume of leach solution applied increases slightly for pulse irrigation.

(76) $$\frac{\partial \left(\theta C_{i}\right)}{\partial t}-\nabla \left(\theta D_{i,jk}\cdot \nabla C_{i}\right)+\nabla \left(qC_{i}\right)=S_{i}+S_{i}^{cl}\;|\;S^{cl}=\nabla \left(k\left(h\right)\nabla H\right)$$

Subsequently, in Hoseinian et al. [116] a mathematical modeling method is used to predict the optimum leaching conditions in copper oxide mineral columns by investigating the effects of variables such as column height (*H*), particle size (*PS*), acidity rate (*AR*) and leaching time (*t*) on copper recovery *R(t)*, as shown in Equations (77) and (78). The results of the fitted model show high efficiency in predicting recovery in leach columns.

(77) $$R\left(t\right)=a\left[\ln\left(d\frac{x}{exp\left(c\right)}\right)\right]+b\{\begin{array}{cc} c=1 & for\;PS=0.0254\\ c=171.26\left(PS/H\right)+0.225 & for\;PS>0.0254 \end{array}$$

(78) $$x=\ln\left(\frac{H}{PS}\right)*t*AR\;;\;d=exp\left(28.059H^{-1.807}\right)$$

In Van Staten and Petersen [117], a first-order exchange model (see Equation (29)) and spherical diffusion (see Equation (30)) [60] are compared with published short-term pulse test data, concluding that the former is simpler and more convenient to use, but the latter produced more realistic results over longer leaching periods. While in Hashemzadeh et al. [118,119], chalcocite leaching kinetics using a variable order kinetic model are modeled, which were already successfully applied in the literature [56,120], and whose formulation are provided in Equation (79).

(79) $$\frac{dx}{dt}=\frac{{\left(1-x\right)}^{\varphi }}{\tau }\;|\;1-x=\{\begin{array}{c} exp\left(\frac{-t}{\tau }\right)@\varphi =1\\ {\left(1-\left(1-\varphi \right)\frac{t}{\tau }\right)}^{\frac{1}{1-\varphi }}@\varphi \neq 1 \end{array}\;\frac{t}{\tau }=\frac{k_{\left(T_{ref}\right)}}{d_{o}^{q}}exp\left(\frac{E_{a}}{R}\left(\frac{1}{T_{ref}}-\frac{1}{T}\right)\right)$$

The two-stage dissolution dynamics of chalcocite generates a different kinetic model for each stage [118,119]. The first stage was controlled by ferric diffusion through the product layer, while the second stage was controlled by mineral decomposition and ferric reduction, which was sensitive to temperatures with high activation energy. Based on the kinetic models, the authors concluded that the leaching rate of the first stage was controlled by ferric ion diffusion, while mixed kinetics governed the kinetics of the second stage, i.e., a combination of mineral decomposition and ferric reduction. Similarly, but applied to modeling the recovery of copper and zinc, Zhang et al. [121] model the leaching rate as $\alpha =\frac{\left[M\right]V}{m\omega }\times 100\%$, where *e* [*M*] is the metal concentration in g/L, *V* is the volume of the leaching solution in L, *m* is the quantity of the materials in g and *ω* is the content of metal in the materials in percentage.

In addition to chalcopyrite leaching, Winarko et al. [122] developed a kinetic model of iodine-assisted chalcopyrite leaching in ferric sulfate media, and selected the shrinking core chemical reaction-controlled model (see Equation (37)) to describe the leaching kinetics of chalcopyrite in the presence of iodine.

Applying the same framework of Aguirre et al. [106], Toro et al. [15] apply the response surface methodology (RSM) to evaluate the effect of three independent variables (time, H_2_SO_4_ and chloride concentration) on the leaching of pure chalcocite to extract copper, fitting a quadratic model that allows to predict extraction. Saldaña et al. [123] develop an experimental design both to evaluate the impact of dependent variables on the response, and to generate analytical models (through multiple regressions) that represent the copper and manganese extractions. Pérez et al. [20] applied the surface optimization methodology using a central composite face design to evaluate the effect of leaching time, chloride concentration and sulfuric acid concentration on the level of copper extraction from covellite. The ANOVA developed by Pérez et al. [20] indicated that leaching time and chloride concentration have the most significant influence, while copper extraction was independent of sulfuric acid concentration. The experimental data was described using a quadratic model.

Additional applications of RMS to copper leaching modeling were found [124,125]. Nozhati and Azizi [124] investigate the leaching behavior of zinc and copper using the RSM model, examining the synergistic and individual effects of five main factors: liquid/solid ratio, sulfuric acid concentration, agitation speed, leaching time and temperature. In Bai et al. [125], the effects of variables such as H_2_SO_4_ concentration, leaching temperature and leaching time, on leaching efficiency are examined. In Sabzezari et al. [126], the RSM and CCD were employed to study the effect of leaching parameters (acid concentration, pulp density, oxidant concentration, microwave power and leaching time) on copper and zinc dissolution. While in Quezada et al. [127], non-linear regression was modeled to represent the dissolution of black copper oxides from residue leaching, as a function of Eh and time.

There are several works in literature where SCM models were applied in recent years [128,129,130,131], for example, Nadirov et al. [128] model copper ammonia leaching from smelter slag, studying the effect of experimental factors (leaching duration, reagent concentration, temperature, agitation rate, as well as a solid-liquid ratio) on copper extraction; Tang et al. [130] develop a kinetic study on metal leaching mechanisms from the upper surface layer of copper aluminates and copper ferrites; Hosseinzadeh et al. [129] model the copper dissolution process from the crushing circuit rejects of a copper heap leaching. The results indicated that the dissolution rate could be controlled by both the chemical reaction and the diffusion process, though the diffusion process was the dominant mechanism in the investigated system, where *C*, *S*/*L*, *n*, and *d_p_* represent the reagent concentration, solid-to-liquid ratio, stirring rate and particle size, respectively. Trinh et al. [131], model the selective recovery of copper by acid leaching from waste sludge. Ambo et al. [132] model the selective leaching of copper from preconcentrated copper ores based on near-infrared sensors, revealing that the rate of leaching increases with increasing ammonium chloride concentration, temperature, decreasing particle size of the ore, the speed of agitation and the solid-liquid ratio. Lee et al. [133] use the SCM model to study the effect of mechanical activation on copper leaching from copper sulfide, CuS, by analyzing the leachability and apparent activation energy. Shi et al. [134], study the kinetics of copper extraction from foundry slag by pressure oxidative leaching with sulfuric acid, adjusting a kinetic equation of leaching. Zhang et al. [135], applied the SCM model to study the leaching behavior of copper and iron recovery from reduction roasting pyrite cinder. It was shown that the leaching process was controlled by mixed diffusion and chemical reaction, which indicated that the leaching rate was controlled by the lixiviant diffusion and surface reaction simultaneously, while that residues characterization indicated that free copper oxide, combined copper oxide and secondary copper sulfide almost completely dissolved in the H_2_SO_4_ solution; however, chalcopyrite only partially dissolved due to the difficulty for H_2_SO_4_ to leach copper (in the form of primary sulfides) at atmospheric pressure. Finally, Apua and Madiba [136] carry out an experimental investigation on the study of the leaching kinetics of copper oxide minerals, investigating the effect of time, pH, stirring speed and temperature on the extent of dissolution, fitting a potential function that explains the recovery of copper (and other metals) over time.

The modeling of copper leaching dynamics was studied in detail by many authors in the generation of representative analytical models of copper extraction/concentration at industrial processes, such as the modeling of extraction dynamics at laboratory level, which is developed with the theoretical aim of contributing to increasing the body of knowledge on the subject. Within the development of models, the most used to model the copper leaching dynamics (and the leaching of other minerals) are the shrinking core models (SCM) and the progressive conversion models (PCM), kinetic models used to analyze the processes kinetics in which chemical reactions take part.

As indicated above and evidenced through the development of this review, there are two mathematical approaches that allow to explain mineral leaching kinetics through simplifications and/or assumptions that conceptually represent the evolution over time of solid interaction (solute), with the leaching liquid (solvent) and the transformation of part of the solute to a product that remains dissolved in the liquid phase. In the progressive conversion approach, a continuous and progressive reaction occurs throughout the ore particle, whereas in the unreacted core model, the reactions proceed in stages and the ore reduces in size as progresses the mineral leaching process. Although the above models are the most widely used in the literature, mathematical formulations were also found, such as inverse exponential models, potential models or multiple regression (linear and non-linear) models, aimed at explaining the concentration or extraction of copper, depending on independent variables of each experimental design. It should be noted that comprehensive kinetic models require more complete knowledge of the dynamics and/or variables involved in the process (observations of these must be available), while mathematical models such as regressions are more useful when looking for a model and simulate the dynamics of the response against variations of the factors to intervene in the experimental design.

To summarize, mineral leaching process modeling contributes to generating a better understanding of the process dynamic through an abstraction of its operation and expressing the mathematical functions that represent its behavior in an integral way. The different models developed in the literature have also contributed to identifying the impact of the variables and/or operational parameters on the copper minerals leaching, and new approaches, such as the application of machine learning techniques [137,138], could lead to significant improvements in the study of the inherent dynamics in mineral processing, or in the generation of systems that support the mineral leaching process [139,140].

## 4. Conclusions and Future Perspectives

Leaching is a process widely used in extractive metallurgy where a mineral is treated with chemicals (solvents) to convert the valuable metals within into soluble salts, allowing the separation of minerals, which in industrial terms translates into the economic exploitation or marginal deposits. At an industrial scale, the main efforts have focused on the acid leaching of copper oxides, uranium minerals and cyanide leaching of gold minerals, and lately, leaching copper sulfide minerals by H_2_SO_4_ + chlorides. The mineral leaching process was developed by many authors, modeling the process mainly applied on an industrial scale of metallic mining of copper. Nevertheless, the trend in leaching processes points to the leaching of copper sulfide minerals (mainly using industrial applications) and to leaching processes that are environmentally friendly. These include the efficient use of water resources, an issue of special interest when considering novel paradigms such as smart industry, circular economy or green economy and the impact of production processes on the carbon footprint.

The first mathematical models that represent the mineral leaching process consider that the process can be represented by an inverse exponential function (a first-order rate equation), which is useful to represent the metallic mineral leaching process, considering the mineral leaching at particle level or modeling the recovery of this at an aggregate level, a set of particles or heap. The models represent leaching kinetics through changes at the physical and chemical level in the individual particles generated, highlighting two models, the shrinking core model (SCM), and the progressive conversion model (PCM). The SMC model considers that the reaction firstly occurs in the outer skin of the particle, leaving a completely converted material and an inert unreacted solid core, while the PCM model considers that the fluid enters and reacts at different rates inside the particle, that is, the solid reagent is converted or reacts continuously and progressively.

In addition to the previous models, modifications incorporate the diversity of particle shapes, industrial scale aggregate models, models that consider the process as a complex system involving variables such as temperature or multiplicity or the reagents and reactants or emerging technologies such as computational fluid dynamics, among others. These models show good performance representing leaching dynamics, both at laboratory and industrial scales (sustained in the generation of huge amounts of data derived from industry 4.0 paradigm). The process complexity and the diversity of its applications, together with the arrival of new technologies, such as machine learning (to be addressed in future reviews), can contribute to generate better models, making the extraction processes more efficient and contributing to improving the economic performance of mineral worksites. Additional mathematical fits of the leaching process are factorial models or the design of experiments (more common in laboratory scale models or industrial scale proof of concept), which rather than contributing to developing models that comprehensively represent the system, allow to generate useful representations when obtaining knowledge in front of intentional alterations over process operating parameters (through techniques as regressing modeling or hypothesis testing).

Finally, the long-term success of industrial applications and technologies such as mineral leaching requires an interdisciplinary approach to the research, the development of new leaching methodologies in the laboratory and the development of improvements in the effectiveness, efficiency and scaling up to the industrial level, while maintaining a focus on sustainability, care for the environmental, preservation of the biodiversity and respect for human rights.

## Figures and Tables

**Table 1:** Review of leaching application to some metallic minerals.

Metallic Mineral	Publications
Copper, Gold, Silver, Uranium	Padilla et al. [29]
Zinc	Qin et al. [30,31]; Petersen and Dixon [32]
Nickel	McDonald and Whittington [33]; Oxley et al. [34]; Khalezov et al. [35]
Platinum	Mwase et al. [36,37,38]; Schoeman et al. [39]
Manganese	Krebs and Milligan [40]; Baumgartner and Groot [41]

**Table 2:** Kinetics models suggested for the leaching process (*X* = fraction reacted, *k* = kinetic constant).

Model	Mechanism	Equation	Reference
$k=1-{\left(1-X\right)}^{1/3}$	Chemical reaction control	(37)	[59]
$k=1-\frac{2}{2}X-{\left(1-X\right)}^{2/3}$	Diffusion control	(38)	[59]
$k=1-{\left(1-0.45X\right)}^{1/3}$	Surface chemical reaction by shrinking core model	(39)	[62]
$k={\left[1-{\left(1-X\right)}^{1/3}\right]}^{2}$	Diffusion through product layer	(40)	[63]
$k=1-\frac{2}{2}X-{\left(1-X\right)}^{1/3}$	Diffusion through a porous product layer by shrinking core model	(41)	[64]
$k=\frac{1}{3}ln\left(1-X\right)+{\left(1-X\right)}^{1/3}-1$	Interfacial transfer and diffusion across the product layer	(42)	[65]
$k=1-3{\left(1-X\right)}^{2/3}+2\left(1-X\right)$	Diffusion of hydrogen ions through a product layer by shrinking core model	(43)	[66]
$k=1-{\left(1-X\right)}^{2/3}$	Mixed control model by shrinking core model (diffusion control; chemical reaction control)	(44)	[67]
k=−ln(1−X)	Mixed control model (surface reaction control; and diffusion through sulfur layer)	(45)	[68]
$k=\frac{1}{5}{\left(1-X\right)}^{-5/3}-\frac{1}{4}{\left(1-X\right)}^{-4/3}+\frac{1}{20}$	Mixed control model based on reactant concentrations	(46)	[69]

## Data Availability

Data sharing not applicable—no new data was generated.
